# Supplementary material for: Evaluation of the National cervical cancer surveillance program in Bangladesh: Performance, strengths, and opportunities for improvement
Source: PLOS Glob Public Health. 2025 May 9;5(5):e0004595. doi: 10.1371/journal.pgph.0004595 (PMC12064025; doi:10.1371/journal.pgph.0004595)
Supplement: S1 Table — (DOCX) [file pgph.0004595.s001.docx]

**Supplementary Table I: The Data Completeness of the NCCSS in Bangladesh, 2023**

| **Variable Name** | **Total** | **Missing** | **Missing Percentage** | **Rank*** |
| --- | --- | --- | --- | --- |
| **District** | 46,383 | 229 | 0.49 | **Excellent** |
| **Education** | 46,383 | 10453 | 22.53 | **Poor** |
| **Family Monthly Income** | 46,383 | 11175 | 24.09 | **Poor** |
| **Husband Living Status** | 46,383 | 10171 | 21.92 | **Poor** |
| **NID** | 46,383 | 0 | 0 | **Excellent** |
| **Occupation** | 46,383 | 0 | 0 | **Excellent** |
| **Parity** | 46,383 | 10484 | 22.60 | **Poor** |
| **Phone number** | 46,383 | 0 | 0 | **Excellent** |
| **Screening Result** | 46,383 | 695 | 1.49 | **Excellent** |
| **Union** | 46,383 | 8852 | 19.08 | **Poor** |
| **Upazila** | 46,383 | 229 | 0.49 | **Excellent** |
| **Patient Visit Date** | 46,383 | 0 | 0 | **Excellent** |
| **Enrollment Date** | 46,383 | 0 | 0 | **Excellent** |
| **Biopsy** | 6,156 | 4,345 | 70.58 | **Poor** |
| **Colposcopic Findings** | 6,156 | 205 | 3.33 | Very **Good** |
| **Colposcopic Findings Final** | 6,156 | 3,662 | 59.48 | **Poor** |
| **Colposcopy Lesion Size** | 6,156 | 3,514 | 57.08 | **Poor** |
| **Histopathological finding** | 6,156 | 5,862 | 95.22 | **Poor** |
| **Overall Completeness** | **806,099** | **105,729** | **13.11** | Average |

*Excelent= <2%, Very Good= 2-5%, Good= 5-110% , 10-15%= Average", poor=>15% **missing data**
