# Supplementary material for: Evaluation of the National cervical cancer surveillance program in Bangladesh: Performance, strengths, and opportunities for improvement
Source: PLOS Glob Public Health. 2025 May 9;5(5):e0004595. doi: 10.1371/journal.pgph.0004595 (PMC12064025; doi:10.1371/journal.pgph.0004595)
Supplement: S1 Text — (PDF) [file pgph.0004595.s002.pdf]

Government of the People's Republic of Bangladesh
Institute of Epidemiology, Disease Control and Research (IEDCR),
Mohakhali, Dhaka- 1212
Telephone: 8802-222280376, 8802-222298691; FAX: 8802-222280440
Email: info@iedcr.gov.bd; Website: www.iedcr.gov.bd

Questionnaire-1: Cervical Cancer surveillance System evaluation **(For project Director, BSMMU / Chief Health Data Unit, Management Information System )**

Your name:

Designation:

Working place:

Contact Number:

Signature:

Date and time:

1. How long have you been involved in the National Cervical and Breast Cancer Surveillance project
2. Can you provide an overview of the goals and objectives of the surveillance project?

3. How is the data analyzed and utilized for decision-making and policy development?

4. Are there any specific strategies in place to ensure the representativeness of the surveillance data?

5. How do you measure the effectiveness and impact of the surveillance project?

6. How does the surveillance project collaborate with healthcare facilities, screening centers, and other stakeholders?

7. Are there any plans for expanding or enhancing the surveillance project in the future?

8. How do you ensure the quality and accuracy of the collected surveillance data?

9. How is the privacy and confidentiality of the collected data ensured?

10. How do you engage and involve healthcare providers, participant, and other stakeholders in the surveillance project?

11. Can you describe any specific initiatives or programs implemented based on the findings from the surveillance data?

12. Can you describe any efforts made to capture data from diverse populations, including marginalized or hard-to-reach groups?

13. How do you ensure the inclusion of underrepresented populations in the surveillance project?

14. Are there any challenges related to equipment and logistics availability for the surveillance project?

1. Please identify the strengths of the system.

2. Please identify the weaknesses of the system.

3. Please identify the opportunities that we may find in the future using this system.

4. Please identify the challenges that you faced as an operational challenge of this system.

5. Please identify some recommendations for betterment of the system.

**Thank you for your kind cooperation, much appreciated.**

-------------------------------------

IEDCR

Government of the People's Republic of Bangladesh
Institute of Epidemiology, Disease Control and Research (IEDCR),
Mohakhali, Dhaka- 1212
Telephone: 8802-222280376, 8802-222298691; FAX: 8802-222280440
Email: info@iedcr.gov.bd;        Website: www.iedcr.gov.bd

Questionnaire-2: Cervical Cancer Surveillance System evaluation **(For Director/Superintend UHFPO/ Colposcopist/SSN/Statistician/CHCP/Heath Assistant/FWV)**

Your name:

Designation:

Working place:

Contact Number:

Signature:

Date and time:

**Please encircle the correct response and document if any comments ( skip questions if this is not relevant to that person)**

6. **Simplicity**

**1.1 How long have you been working with "" Cervical Cancer Surveillance System"?**

1. Less than 6 months    2. 6 months to 1-year

3. More than 1-year    4.From the beginning of the system launched

**1.2 Do you receive training for Cervical Cancer surveillance"?**

1.Yes    2. No

Comments:

**1.3 Do you think Report forms are available to your center?**

1.Yes    2. No

Comments:

**1.4 Do you think there are any unnecessary steps in the "Cervical Cancer Surveillance System"?**

1.Yes 2. No

Comments:

## 2 Flexibility:

**2.1 Have there been any changes in case definition since the screening center started?**

1.Yes 2. No

Comments:

**2.2 Have there been any changes in the information form/registry since the screening center started?**

1.Yes 2. No

Comments:

**2.3 Do you have access to edit Data for wrong Update?**

1.Yes 2. No

Comments:

**2.4 Do you have access to Change Password of DHIS2?**

1.Yes 2. No

Comments:

**2.5 Have there been any changes in the reporting system since the screening center started?**

1.Yes 2. No

Comments:

## 3. Acceptibility

**3.1 do you think human resources are persistently available/posted in this center?**

1.Yes 2. No

Comments:

## 4. Stability

**4.1** Is equipment and logistics always available for the screening center?

1.Yes 2. No

Comments:

**4.2 In case of outages and power failures, does your center have alternate power supply available?**

1.Yes 2. No

Comments:

**4.3 Does Surveillance provide Computer for Data entry?**

1.Yes 2. No

Comments:

**4.4 Does program provide Internet Facility for Data entry?**

1.Yes 2. No

Comments:

**4.5 Does Surveillance provide of Referral Slip ?**

1.Yes 2. No

Comments:

4.6 Does your center have Availability of Core support from Surveillance ?

1.Yes 2. No

Comments:

**4.7 Does your center have Availability of dedicated team for surveillance?**

1.Yes 2. No

Comments:

**4.8 Do you think the system could adapt easily to fund Variation?**

1.Yes 2. No

Comments:

**5. Usefulness**

**5.1 Does surveillance regularly Generate of Report from Surveillance System?**

1.Yes 2. No

Comments:

**5.2 Does cervical cancer surveillance regularly disseminate Report your center?**

1.Yes 2. No

Comments:

**5.3 Do you think this surveillance is useful for early prevention and management related decision making?**

1.Yes 2. No

Comments:

**5.4 How useful cervical cancer surveillance System?**

| Very useful | Somewhat useful | neutral | not very useful | not at all useful |
|---|---|---|---|---|
| 5 | 4 | 3 | 2 | 1 |

.

Please read the following statements carefully and grade them on the scale according to your experience (circle the chosen number).

**1 = Strongly disagree 2=Disagree, 3=Neutral, 4=agree & 5 = Strongly agree**

**Simplicity**

| | | | | | |
|---|---|---|---|---|---|
| SOP is easily applicable for operational management of surveillance | 1 | 2 | 3 | 4 | 5 |
| Case definition is easy to identify case | 1 | 2 | 3 | 4 | 5 |
| Collecting data do not need much time | 1 | 2 | 3 | 4 | 5 |
| The follow up of case is very easy | 1 | 2 | 3 | 4 | 5 |
| The Flow chart/data flow of the system is simple | 1 | 2 | 3 | 4 | 5 |
| The reporting system is easy | 1 | 2 | 3 | 4 | 5 |

**Acceptability**

| | | | | | |
|---|---|---|---|---|---|
| You are willing to participate in this surveillance | 1 | 2 | 3 | 4 | 5 |
| You are completely satisfied with surveillance system | 1 | 2 | 3 | 4 | 5 |
| Participants s are willing to participate in this surveillance | 1 | 2 | 3 | 4 | 5 |
| Operating experience with existing human resource | 1 | 2 | 3 | 4 | 5 |
| System Assured participant privacy and confidentiality | 1 | 2 | 3 | 4 | 5 |
| A similar system can be considered for future program designing | 1 | 2 | 3 | 4 | 5 |

**Flexibility**

| | | | | | |
|---|---|---|---|---|---|
| You are Adapted to overcome the challenges appeared routinely during system use | 1 | 2 | 3 | 4 | 5 |

| | |
|---|---|
| This system is useful for early prevention & management related decision making | 1 2 3 4 5 |

6 Please identify the strengths of the system.

7 Please identify the weaknesses of the system.

8 Please identify the opportunities that we may find in the future using this system.

9 Please identify the challenges that you faced as an operational challenge of this system.

10 Please identify some recommendations for betterment of the system.

**Thank you for your kind cooperation, much appreciated.**

-------------------------------------

Signature & Date
