# Supplementary material for: Evaluation of the National cervical cancer surveillance program in Bangladesh: Performance, strengths, and opportunities for improvement
Source: PLOS Glob Public Health. 2025 May 9;5(5):e0004595. doi: 10.1371/journal.pgph.0004595 (PMC12064025; doi:10.1371/journal.pgph.0004595)
Supplement: Attachment — Submitted filename: authors response to reviewer comments.docx [file pgph.0004595.s005.docx]

**Manuscript Number: PGPH-D-24-02609**

**Title: Evaluation of the National Cervical Cancer Surveillance Program in Bangladesh: Performance, Strengths, and Opportunities for Improvement**

| **Reviewer/Editor Comments** | **Authors' Response** |
| --- | --- |
| **Reply to editors review** | |
| Please amend your detailed Financial Disclosure statement. This is published with the article. It must therefore be completed in full sentences and contain the exact wording you wish to be published.  **Please only choose the relevant sentences from below**  i. Please clarify all sources of funding (financial or material support) for your study. List the grants (with grant number) or organizations (with url) that supported your study, including funding received from your institution.  ii. State the initials, alongside each funding source, of each author to receive each grant. iii. State what role the funders took in the study. If the funders had no role in your study, please state: “The funders had no role in study design, data collection and analysis, decision to publish, or preparation of the manuscript.” iv. If any authors received a salary from any of your funders, please state which authors and which funders.  2. Please make sure the funding information on the submission form matches your financial disclosure statement. Please indicate by return the full and correct funding information for your study and confirm the order in which funding contributions should appear. Please be sure to indicate whether the funders played any role in the study design, data collection and analysis, decision to publish, or preparation of the manuscript. | We appreciate the opportunity to clarify the financial disclosure statement. This has been amended as follows:  The study did not receive any external grant but was partially supported by the Institute of Epidemiology, Disease Control and Research (IEDCR), Dhaka, Bangladesh (website: www.iedcr.gov.bd). The funders had no role in study design, data collection and analysis, decision to publish, or preparation of the manuscript. Additionally, no authors received a salary from the supporting institution for this study.  Please let us know if any further revisions are required. |
| 3. We have amended your Competing Interest statement to comply with journal style. We kindly ask that you double check the statement and let us know if anything is incorrect. This policy applies to all data except where public deposition would breach compliance with the protocol approved by your research ethics board. If your data cannot be made publicly available for ethical or legal reasons (e.g., public availability would compromise patient privacy), please explain your reasons on resubmission and your exemption request will be escalated for approval. | Thank you for your feedback and for amending the Competing Interest statement to align with the journal's style. I have reviewed the statement and confirm that it is accurate. I appreciate your attention to detail, and everything appears to be in order.  Regarding the data, I would like to confirm that all data used in the manuscript is included and has been properly referenced. The amended Competing Interest statement accurately reflects this, and I trust that it meets the journal's requirements. |
| 4. In the online submission form, you indicated that "The data collected for this study has been analyzed and is included  within this published article. The data analyzed in this study is available on justifiable request.".  All PLOS journals now require all data underlying the findings described in their manuscript to be freely available to other researchers, either  1. In a public repository,  2. Within the manuscript itself, or  3. Uploaded as supplementary information.  This policy applies to all data except where public deposition would breach compliance with the protocol approved by your research ethics board. If your data cannot be made publicly available for ethical or legal reasons (e.g., public availability would compromise patient privacy), please explain your reasons by return email and your exemption request will be escalated to the editor for approval. Your exemption request will be handled independently and will not hold up the peer review process, but will need to be resolved should your manuscript be accepted for publication. One of the Editorial team will then be in touch if there are any issues. | Thank you for outlining the journal’s data availability requirements. We would like to confirm that all data underlying the findings in our manuscript has been fully included within the manuscript and supplementary information. Additionally, we have amended the Data Availability Statement in the online submission form to ensure compliance with the journal’s policy.  Please let us know if any further clarifications are needed. We appreciate your guidance and support throughout the review process. |
| 5. We have noticed that you have uploaded Supporting Information files, but you have not included a list of legends. Please add a full list of legends for your Supporting Information files after the references list. | We added the list |
| 6. We notice that your supplementary table 1 has a manuscript file. Please remove them and upload them with the file type 'Supporting Information'. Please ensure that each Supporting Information file has a legend listed in the manuscript after the references list. | Revised accordingly |
| 7. Figure 2: please (a) provide a direct link to the base layer of the map (i.e., the country or region border shape) and ensure this is also included in the figure legend; and (b) provide a link to the terms of use / license information for the base layer image or shapefile. We cannot publish proprietary or copyrighted maps (e.g. Google Maps, Mapquest) and the terms of use for your map base layer must be compatible with our CC-BY 4.0 license.  Note: if you created the map in a software program like R or ArcGIS, please locate and indicate the source of the basemap shapefile onto which data has been plotted.  If your map was obtained from a copyrighted source please amend the figure so that the base map used is from an openly available source. Alternatively, please pro+vide explicit written permission from the copyright holder granting you the right to publish the material under our CC-BY 4.0 license.  Please note that the following CC BY licenses are compatible with PLOS license: CC BY 4.0, CC BY 2.0 and CC BY 3.0, meanwhile such licenses as CC BY-ND 3.0 and others are not compatible due to additional restrictions.  If you are unsure whether you can use a map or not, please do reach out and we will be able to help you. The following websites are good examples of where you can source open access or public domain maps:  * U.S. Geological Survey (USGS) - All maps are in the public domain. (http://www.usgs.gov)  * PlaniGlobe - All maps are published under a Creative Commons license so please cite “PlaniGlobe, http://www.planiglobe.com, CC BY 2.0” in the image credit after the caption. (http://www.planiglobe.com/?lang=enl)  * Natural Earth - All maps are public domain. (http://www.naturalearthdata.com/about/terms-of-use/) | Thank you for your guidance regarding Figure 2. We confirm that the map was created using QGIS version 3.34, and the base layer (country/region borders) was sourced from GADM, version 4.1, which is available under a Creative Commons Attribution license (CC BY 4.0) and fully compatible with the journal’s licensing requirements.  (a) We have now included a direct link to the base layer source in the figure legend:  (<https://gadm.org/download_country.html#google_vignette>)  (b) The terms of use for GADM data can be found here: GADM License. (<https://gadm.org/license.html>) |
| **Response to Reviewer 1 Comments** | |
| • Abstract background does not identify the research gap in the field, rather elaborates how the research study was conducted. It is important to mention why the research was conducted and how it bridges the gap of exciting knowledge in the field. | Thank you for your valuable feedback. We have revised the Background section of the abstract to clearly highlight the research gap and the significance of our study. While cervical cancer remains a major public health concern in Bangladesh, the Electronic Data Tracking with Population-Based Cervical and Breast Cancer Screening Program (EPCBCSP) has been operational since 2018 without prior evaluation. The lack of systematic assessment limits understanding of the program’s effectiveness, challenges, and areas for improvement. Our study addresses this gap by evaluating the performance, strengths, and limitations of EPCBCSP, providing critical insights to enhance cervical cancer surveillance and screening efforts in Bangladesh. |
| It is unusual to have subheading for different part of the Introduction section. It should have a flow of background knowledge, present condition and treatments of cervical cancer in Bangladesh, current research gap and finally what the current study aims, objectives and brief results summary without any subheading. | Thank you for your valuable feedback. We acknowledge the importance of maintaining a coherent and integrated flow in the Introduction section, rather than using subheadings. However, given that this study is a system evaluation, we structured the introduction to provide a comprehensive understanding of the cervical cancer surveillance system in Bangladesh, including its background, implementation, and challenges, before presenting the research gap and study objectives.  Our approach follows the format of previous surveillance system evaluation manuscripts, ensuring clarity and a logical flow for readers unfamiliar with the EPCBCSP framework. Since this study evaluates an existing surveillance system, it was necessary to present the historical context, implementation process, and reporting mechanisms to establish a foundation for assessing its performance. This background also helps in identifying system gaps and understanding the rationale behind our evaluation.  In response to the reviewer’s concerns, we have removed subheadings while maintaining a structured narrative that flows logically from background knowledge to research gaps and study objectives. We believe this revision enhances readability and aligns with the standards for surveillance system evaluation studies. However, we are open to further modifications based on editorial recommendations. |
| Methods section should detail as much as possible. Since the data were collected via in depth interview using a semi-structured questionnaire adapted from updated CDC guideline, a copy of such questioner should be included in the supplementary document for the evaluation | Thank you for your insightful feedback. We acknowledge the importance of detailing the Methods section for clarity and reproducibility.  To address this:   - We have explicitly mentioned that data were collected through in-depth interviews using a semi-structured questionnaire adapted from the updated CDC guidelines for evaluating public health surveillance systems. - The semi-structured questionnaire has been included as a supplementary document to allow readers to review the specific questions used in stakeholder interviews. - The CDC evaluation guideline was cited in the references as it provides comprehensive details on the evaluation framework. To maintain brevity and avoid redundancy, we did not include the full guideline details in the manuscript. However, interested readers can refer to the original guideline for an in-depth understanding. - We have also elaborated on the study design, participant selection, data collection, and scoring methodology to ensure clarity while maintaining a concise presentation.   We appreciate the reviewer’s recommendation and believe these revisions enhance the methodological transparency of our study |
| • No statistical analysis of the data was done. Please select an appropriate statistical analysis such as confidence interval for the data. | Thank you for your valuable feedback.  Our study aimed to evaluate the National Cervical Cancer Surveillance System (NCCSS) using a descriptive, cross-sectional approach based on the updated CDC guidelines. Given the nature of this evaluation, we focused on descriptive analysis rather than inferential statistical methods.  The quantitative data collected from structured interview responses were summarized using summary statistics (percentages, frequencies) to describe stakeholder perceptions of system attributes.  The qualitative data were analyzed narratively to capture themes and insights from stakeholder responses.  The scoring system applied to surveillance system attributes (e.g., simplicity, flexibility, data quality) was based on percent score calculations, which allowed classification into performance categories (Excellent, Very Good, Good, etc.).  As our study did not involve hypothesis testing or comparisons between groups, confidence intervals and other inferential statistical analyses were not applicable. However, if necessary, we can provide additional statistical summaries to further enhance the descriptive findings.  We appreciate the reviewer’s suggestion and are open to incorporating additional descriptive statistical measures if required. |
| Results section must highlight result-oriented subheadings and section rather than adjective titles. | Thank you for your helpful comment regarding the subheadings in the results section.  The organization of the results section follows the structure used in previous evaluation studies, such as those by Laryea et al. (2022) in Ghana and Al-Jamrah et al. (2021) in Yemen, to maintain consistency and clarity in presenting the findings. These studies used subheadings that focused on specific components of the evaluation, which we have similarly adopted to assess various surveillance system attributes like data quality, flexibility, acceptability, stability, and usefulness. This approach aligns with established evaluation practices and provides a clear presentation of the study outcomes (Laryea et al., 2022; Al-Jamrah et al., 2021).  We believe this structure enhances the readability of the results and ensures they are aligned with previous evaluations in the field. Thank you once again for your feedback.  References:   - Laryea EB, Frimpong JA, Noora CL, Tengey J, Bandoh D, et al. (2022). Evaluation of the adverse events following immunization surveillance system, Ghana, 2019. *PLOS ONE*, 17(3): e0264697. <https://doi.org/10.1371/journal.pone.0264697> - Al-Jamrah KM, Al Nabehi BA, Almoayed KA, Anam LS, Khader YS. (2021). Performance of the Neonatal Tetanus Surveillance System (NTSS) in Sana'a, Yemen: Evaluation Study. *JMIR Public Health Surveill*, 7(5):e27606. doi:10.2196/27606 |
| Similar to Introduction section, the Discussion section should not have any subheadings or highlights. | Highlight’s removed |
| Conclusion section should concisely summarize the Bangladesh's NCCSS and it’s limitations. | Conclusion was revised accordingly |
| English throughout the manuscript is erroneous and thus difficult to understand. | Thank you for your valuable feedback. We acknowledge the language issues in the manuscript and have carefully revised the text to improve clarity, coherence, and readability. |
| **Response to Reviewer 2 Comments** | |
| Introduction CC is not an acceptable synonym for cervical cancer. | Thank you for your feedback. We acknowledge the concern regarding the abbreviation "CC" for cervical cancer. However, "CC" has been used as a recognized abbreviation in several peer-reviewed publications, including those cited (<https://pmc.ncbi.nlm.nih.gov/articles/PMC10624144/> and <https://onlinelibrary.wiley.com/doi/abs/10.1002/ijc.10453>). Given its usage in scientific literature, we believe it is appropriate within the context of our study. However, if the editorial team prefers, we are willing to replace "CC" with "cervical cancer" throughout the manuscript for clarity and consistency |
| Authors begin the sentence (Page 3, Line No: 82) as CC, which is never acceptable. | CC was replaced with Cervical cancer |
| Authors have to get Ethical clearance from Institutional Research Board. Ethical clearance Number should be mentioned. | Ethical statement revised with letter from IRB |
| Authors should add limitations of VIA They should discuss the latest recommended method for cervical cancer screening (HPV DNA testing) | Thank you for your suggestion. Our study focuses on evaluating the National Cervical Cancer Screening System (NCCSS) rather than assessing the effectiveness of specific screening methods. While we acknowledge the limitations of VIA and the global shift towards HPV DNA testing as the preferred screening method, our primary objective was to assess the performance and attributes of the surveillance system itself |
| **Response to Reviewer 3 Comments** | |
| Thank you for inviting me to review this important and relevant manuscript and analysis evaluating the Electronic Data Tracking with Population Based Cervical and Breast Cancer Screening Program (EPCBCSP) in Bangladesh  In general, it needs to be clear from title that this is qualitative evaluation of the surveillance systems through interviews with key stakeholders involved in the surveillance programme, and not an evaluation of the cervical cancer screening and treatment programme itself. The current title is a bit misleading.  In general, the presentation of results needs some simplification. Currently, authors are assuming that the reader has a detailed understanding of the CDC methodology for evaluating surveillance . More work is needed to explain in methods, the individual outcomes evaluated, and the results section need to detail what the numerators and denominators refer to. | Thank you for your insightful feedback. We have revised the title to accurately reflect that this study is a qualitative evaluation of the National Cervical Cancer Surveillance System (NCCSS) rather than an assessment of the screening and treatment program. The updated title is: "Evaluation of the National Cervical Cancer Surveillance Program in Bangladesh: Performance, Strengths, and Opportunities for Improvement."  Regarding the methods section, we have clearly outlined that this is a cross-sectional descriptive evaluation study based on the updated CDC guidelines for public health surveillance system evaluation. The study design, sampling, and data collection methods have been explicitly described to ensure clarity for readers unfamiliar with the CDC framework. We have also provided a detailed explanation of the six surveillance system attributes assessed and the scoring methodology used for quantitative and qualitative analyses.  In our evaluation, **numerators and denominators** were clearly defined for each metric. For **data quality**, the numerator was the number of missing or incomplete entries in DHIS2, while the denominator was the total expected data entries. For **qualitative attributes** (simplicity, flexibility, acceptability, stability, and usefulness), the numerator was the sum of stakeholder responses on a Likert scale, and the denominator was the maximum possible score. The **overall system performance** was calculated as the sum of attribute scores divided by the total number of attributes. |
| In methods, authors refer to “CDC guideline for evaluating public health surveillance system” Authors should provide definitions for each of the outcomes reported : simplicity, flexibility, acceptability, and stability, and usefulness. How were each of these outcomes defined and measured? For example: the outcome of acceptability - is this referring to acceptability of screening to the woman undergoing screening or the healthcare provider providing the service? Or is it acceptability of the surveillance system at healthcare provider level? Further information is also needed on how the attribute is qualified, i.e. what is the gold standard for that particular attribute (how is the maximum score assigned? what does maximum score look like? ) As it was difficult to understand what attributes of the cervical cancer screening programme are being evaluated, it was difficult to interpret the findings reported. | As mentioned in the **Methods** section, the definitions and outcomes of the attributes (simplicity, flexibility, acceptability, stability, and usefulness) were detailed in the **CDC guidelines**. However, to avoid redundancy, we did not provide extensive details here. The scoring system was aligned with previous studies for consistency. |
| Line 226 – The numerator and denominator ((1177/1284) need to be explained. What do these numbers refer to ? Individuals interviewed, clinics? | The numbers (1177/1284) refer to the total scores obtained for the attribute of simplicity (1177) out of the maximum possible score (1284). These scores were derived from responses gathered from the stakeholders interviewed as part of the evaluation. The numerator (1177) represents the total score accumulated across all respondents for the simplicity attribute, while the denominator (1284) represents the maximum possible score that could be achieved based on the number of respondents and the Likert scale used for the scoring. This calculation was performed using the methods outlined in the methodology section. |
| Lin 228: “Case definition is easy to identify a case’. Does a case refer to cervical cancer ,or cervical precancerous lesions? | The case in this context refers to whether a case is easily identified according to the definition provided by the program. Specifically, it refers to the clarity and ease with which the surveillance system can identify individuals who meet the program's criteria for cervical cancer or precancerous lesions. The definition used by the program serves as the guideline for identifying and reporting cases. |
| Line 230: 'Follow-up of cases is easy – again is this follow-up of women with cervical precancer or cervical cancer. How a case is defined is important because the ease of follow-up will be very different depending on whether it is precancer or cancer | The follow-up conducted is in accordance with the program's guidelines. This question reflects whether the follow-up process is perceived as easy according to the program's definition, which includes both cervical cancer and cervical precancer cases as per the program's criteria. |
| Table 1 – it is not clear how the column “score” is quantified. How Is “score percent” calculated? How does it relate to the earlier figures in line 226  (1177/1284) ?  Table 1- footnote b refers to Likert scale responses, but this is not explained in the methods | The score percent is calculated based on the method detailed in the methodology section. Specifically, it is derived from the sum of the scores of each indicator divided by the maximum possible score for that indicator, multiplied by the number of respondents. The formula is:  Score Percentage  =  Sum of all respondent’s scores for each indicator  Max score of indicator  ×  No. of respondents  ×  100  Score Percentage=  Max score of indicator×No. of respondents  Sum of all respondent’s scores for each indicator    ×100  The numbers (1177/1284) in line 226 refer to the total scores accumulated by all respondents, and these were used to calculate the overall performance and score percentages for each attribute in Table 1.  The use of Likert scale responses was detailed in the methods section, where we explained how stakeholders rated various attributes using a 5-point Likert scale (1 = strongly disagree; 2 = disagree; 3 = neutral; 4 = agree; 5 = strongly agree). The Likert scale was employed to measure the qualitative attributes (e.g., simplicity, flexibility, acceptability, etc.) based on the level of agreement or disagreement from the stakeholders. |
| A more detailed description of the stakeholders interviewed would be helpful – does it include individuals actively involved in the surveillance of screening and treatment? In what capacity? (number/% health care provider , data cleaning/analysis, interpretation, high level stakeholders etc.) | A detailed description of the stakeholders interviewed is provided in the methodology section, where we specify the roles of individuals involved in the surveillance system, such as hospital directors, UHFPOs, nurses, colposcopists, Community Healthcare Providers (CHCPs), Health Assistants, and key personnel from central-level agencies like the EPCBCSP and DGHS. The stakeholders were chosen based on their direct involvement in the surveillance and screening program. |
| Can authors confirm the sentence “ All ever-married women over the age of 30 made up the target group for CC screening,. What of unmarried women over the age of 30 years? I refer to the National Strategy for Cervical Cancer Prevention (2017-2022) which states that “The target population for screening will be women between 30-60 years of age; and the screening interval will be 5 years. “ | The program did not enroll unmarried women over the age of 30 for cervical cancer screening. This clarification has been added, and a reference to the National Strategy for Cervical Cancer Prevention (2017-2022), which specifically targets women aged 30-60 for screening, has also been included for further clarity. |
| page 4, line 112: there are too many abbreviations, suggest simplifying for ease of reading “specialist group represented by DGHS, 112 DGFP, NICRH, BSMMU, OGSB, WHO, UNFPA, UNICEF and approved by MOHFW [12]” | The sentence has been revised for clarity, simplifying the list of abbreviations to make the text more readable and understandable. |
| line 119: could authors clarify the level of health facility referred to “across 601 health facilities covering all subdistricts in Bangladesh”, i..e what population do these 601 health facilities represent – urban, semi-urban, rural? | The 601 health facilities mentioned encompass both urban, semi-urban, and rural populations across Bangladesh. This has been clarified in the revised text. |
| Lines 206-224: It’s not clear if this paragraph details a finding from the interviews or background information on the screening and surveillance programme. If the latter, it should be given in the introduction | The paragraph in question refers to findings from interviews with central stakeholders. This is mentioned in title that this information is from central stakeholder interview |
